# Fermentation performance and physiology of two strains of *Saccharomyces cerevisiae* during growth in high gravity spruce hydrolysate and spent sulphite liquor

**DOI:** 10.1186/1472-6750-14-47

**Published:** 2014-05-21

**Authors:** Emma Johansson, Charilaos Xiros, Christer Larsson

**Affiliations:** 1 Department of Chemical and Biological Engineering, Chalmers University of Technology, 412 96 Göteborg, Sweden; 2 SP Processum AB, 891 22 Örnsköldsvik, Sweden

**Keywords:** Lignocellulosic material, Nutrients, Energy charge, Fermentation capacity, High gravity fermentation

## Abstract

**Background:**

Lignocellulosic materials are a diverse group of substrates that are generally scarce in nutrients, which compromises the tolerance and fermentation performance of the fermenting organism. The problem is exacerbated by harsh pre-treatment, which introduces sugars and substances inhibitory to yeast metabolism. This study compares the fermentation behaviours of two yeast strains using different types of lignocellulosic substrates; high gravity dilute acid spruce hydrolysate (SH) and spent sulphite liquor (SSL), in the absence and presence of yeast extract. To this end, the fermentation performance, energy status and fermentation capacity of the strains were measured under different growth conditions.

**Results:**

Nutrient supplementation with yeast extract increased sugar uptake, cell growth and ethanol production in all tested fermentation conditions, but had little or no effect on the energy status, irrespective of media. Nutrient-supplemented medium enhanced the fermentation capacity of harvested cells, indicating that cell viability and reusability was increased by nutrient addition.

**Conclusions:**

Although both substrates belong to the lignocellulosic spruce hydrolysates, their differences offer specific challenges and the overall yields and productivities largely depend on choice of fermenting strain.

## Background

Lignocelluloses are a diverse group of substrates [1] including cellulose, hemicellulose, lignin and extractives [2], which produce inhibitors during hydrolysis. The quantity of inhibitor depends not only on the origin of the material but also on the pre-treatment and hydrolysis method. Fermentation inhibitors, which inhibit yeast metabolism, include 5-hydroxymethyl furfural (HMF), 2-furaldehyde (furfural), phenolic compounds and weak acids for review, see [3-6]. Reportedly, weak acids exert a growth inhibitory effect by inflow of non-dissociated acid into the cytoplasm of the microorganism [7]. HMF and furfural are consumed by *S. cerevisiae*, with consequent cost of ATP [8,5]. The inhibitory effect of phenolic compounds on yeast metabolism remains under investigation. A feasible suggestion is that phenolic compounds degrade the cell membrane integrity, reducing the membrane’s efficacy as a selective barrier [9]. To release high amounts of monosaccharides for fermentation, lignocelluloses (on account of their recalcitrant nature) often require harsh pre-treatment conditions. In most cases, the quantity of inhibitors increases with the severity of pre-treatment. Therefore, scientists seek mild pre-treatments that maximize the saccharification yields.

Besides containing growth inhibitors, lignocelluloses are generally scarce in nutrients and nutrient supplementation is thought to increase fermentation performance [10-12]. Earlier studies on such raw materials have identified lack of nitrogen as a crucial limiting factor in fermentations. Nitrogen is especially important for fermentative performance because it promotes cell proliferation [13,14] and thereby ethanol yields, since the ethanol production rate is maximized in actively growing cells [15,16].

Efficient distillation energy requires both high productivity and high ethanol titres. A prerequisite of ethanol production is high-gravity substrate, which theoretically yields at least 40–50 g L^−1^ ethanol [17]. In practice, however, increasing the initial high dry matter content will also increase the concentrations of inhibitory compounds. The tolerance to inhibitors and the fermentation performance of cells depends on the nature of the substrate and the extent to which the fermenting organism is adapted to the specific challenges imposed by the substrate [18,19]. Substrate stress affects the energy metabolism of the yeast cells, since many stress responsive processes depend on ATP availability [20]. Some of the inhibitory compounds in lignocellulosic material appear to decrease the specific sugar uptake rate and the specific ethanol production rate [21,5], both of which are highly correlated with ATP production. Adenine nucleotides also participate in numerous intracellular reactions, and their intracellular concentrations may greatly affect metabolism and yeast cell performance [22]. The study of energy metabolism can therefore provide insights into the maintenance requirements of yeast cells in lignocellulosic fermentations.

To determine the re-usability of the yeast cells, we must know whether the cells can sustain high ethanol production rates under extended periods of stress and nutrient limitation. This is crucial for processes involving cell recycling or re-circulation. During cultivation, the ethanol production rate may not reflect the ethanol production capacity, because cell performance is reduced by the abovementioned substrate limitations. In this study, we measured the fermentation capacity of the cells after re-inoculation in a non-inhibitory nutritionally rich medium. Using this approach, we can detect whether the cells are irreversibly affected by the previous fermentation conditions.

To evaluate the different performance of yeast strains grown in different lignocellulosic substrates and the effect of nutrient addition, the fermentation performance, energy status and fermentative capacity of the strains were measured.

## Methods

### Microorganisms

Two yeast strains were used: *Saccharomyces cerevisiae* Thermosacc (Thermosacc) (Lallemand, USA) and *Saccharomyces cerevisiae* CCUG 53310 (CCUG) (Culture Collection University of Gothenburg, Sweden). Thermosacc is a commercial thermotolerant strain of *S. cerevisiae* developed to withstand the stress of industrial fermentation and higher concentrations of organic acids [23]. CCUG is an industrially harvested yeast strain, selected because it originates from spent sulphite liquor ethanol plant.

### Media and chemicals

The fermentation media were a filtrated spruce dilute-acid hydrolysate (SH) with a water-insoluble solids (WIS) content of 20% (kindly provided by SEKAB E-Technology) and spent sulphite liquor (SSL) (kindly provided by Domsjö Fabriker, Aditya Birla). The concentrations of available hexoses, weak acids, HMF, furfural and phenolics in the spruce hydrolysate were 66.3 g L^−1^, 8.0 g L^−1^, 2.4 g L^−1^, 1.9 g L^−1^ and 5.0 g L^−1^respectively. In the spent sulphite liquor, the respective concentrations of the same compounds were 35.6 g L^−1^, 6.4 g L^−1^, 0.3 g L^−1^, 0.2 g L^−1^ and 1.0 g L^−1^. The spruce hydrolysate slurry was centrifuged and the supernatant was filtered through 0.2 μm pore size filters. The spent sulphite liquor was used without centrifugation and filtration. All chemicals were of analytical grade and were purchased from Sigma Aldrich (Sweden). Yeast extract was purchased from Becton Dickinson (Sweden).

### Cell cultivations and fermentations

Cells were proliferated under aerobic conditions in 250 ml Erlenmeyer flasks using a defined media containing excess nutrients and vitamins [7]. The flasks were incubated at 30°C in an orbital shaker at 180 rpm. At the end of the aerobic growth phase (when the glucose was consumed) SH or SSL was added to the culture to a final concentration of 25% of the initial concentration (final volume ratio in the culture 1:4). The cultures were again aerobically incubated until all glucose was consumed. The fermentations were performed in batch mode in 300 ml Erlenmeyer flasks equipped with a glycerol loop to release CO_2_ and exclude oxygen. The temperature was set to 30°C and agitation to 150 rpm. Prior to inoculation and filtration, the pH of all cultivations was adjusted to 5.5 with 5 M NaOH. As nutrient supplement, 1% (w/v) yeast extract was supplied according to the experimental design.

All experiments were performed in duplicate. CCUG is a flocculating strain, which affects the reproducibility of its results [24].

### Fermentation capacity tests

In the fermentation capacity tests, a 10 ml sample was withdrawn from the lignocellulosic fermentation, and the cells were pelleted by centrifugation (SIGMA Laborzentrifugen GmbH, Osterode, Germany) at 4°C. The pelleted cells were inoculated in a nutritionally rich media containing 20 g L^−1^glucose, 20 g L^−1^ peptone and 10 g L^−1^ yeast extract. Throughout the next 60 min, samples were regularly withdrawn (t = 10, 20, 40 and 60 min) and their ethanol content determined.

### Cell viability test

Cell viability was evaluated by enumerating the colony forming units (CFU). Cells were grown on nutritionally rich non-inhibitory agar plates containing 20 g L^−1^ glucose, 20 g L^−1^ peptone and 10 g L^−1^ yeast extract.

### Extraction of ATP, ADP, AMP and measurements of energy status

Samples (3 ml) were taken for ATP, ADP and AMP measurements and quenched as described in [25] in 17 ml pure methanol maintained at −40°C. The cells were pelleted in a centrifuge (SIGMA Laborzentrifugen GmbH, Osterode, Germany) at −20°C, 4000 *g* for 5 min, flash-frozen in liquid nitrogen and stored at −80°C until analysis. The ATP, ADP and AMP were then extracted according to [26]: 0.5 ml of 0.51 M trichloroacetic acid (TCA) containing 17 mM EDTA was added and the samples were incubated at 4°C for 15 minutes. The extracts were then centrifuged at 18078 *g* for 3 min and subsequently neutralized with 2 M Tris-base.

The energy charge was calculated as follows [27]:

$$\mathrm{EC}=\left(\left(\left[\mathrm{ATP}\right]+\frac{1}{2}\left(\left[\mathrm{ADP}\right]\right)\right)/\left(\left[\mathrm{ATP}\right]+\left[\mathrm{ADP}\right]+\left[\mathrm{AMP}\right]\right)\right).$$

### Analytical procedures

The media were analysed by MoRe research using an Aminex HPX-87H column maintained at 45°C. Glycerol, ethanol and organic acids were detected by an RI detector, and a UV detector was used for HMF and furfural. The eluent was 10 mM H_2_SO_4_ and flow rate was 0.8 ml min^−1^.

After the fermentation, ethanol and glycerol were analysed by HPLC using an RI detector and an Aminex HPX-87H column with a 30 mm × 4.6 mm Cation-H Bio-Rad micro-guard column maintained at 45°C. The eluent was 5 mM H_2_SO_4_ and flow rate was 0.6 ml min^−1^. Hexoses were analysed by high performance anion exchange chromatography using an electrochemical detector and a 4 mm × 250 mm Dionex CarboPac PA1 column with a 4 mm × 50 mm guard column maintained at 30°C. Elution was performed at 1 ml min^−1^ using eluents A (300 mM NaOH) and B (100 mM NaOH + 85 mM sodium acetate). The adenine nucleotides were analysed by HPLC (Ultimate 3000, Dionex Corp., Sunnyvale, US) with an Luna® 5u C18(2) 100 Å LC column (150 mm × 4.6 mm) (Phenomenex Inc., Torrance, US) maintained at 20°C. The mobile phase was acetonitrile and tetrabutylammonium buffer (0.005 M tetrabutylammonium hydrogensulfate, 0.01 M Na_2_HPO_4_), pH 7.0. The acetonitrile gradient was as follows: t0 min 6%, t3 min 6%, t16 min 25%, t22 min 25%, t27 min 6%. The system was then equilibrated for 8 minutes to the initial conditions. The flow rate was 1 ml/min. All detections were performed with a photodiode array detector PDA-3000 (Dionex Corp., Sunnyvale, US) operated at 260 nm. Peak identities were confirmed by co-elution with standards and quantified by comparison with standard solutions of known concentrations.

## Results and discussion

This study investigated the effects of fermenting strain, fermentation substrate and nutrient supplementation of the medium on the fermentative performance and physiological characteristics of *S. cerevisiae*. Effects were evaluated by ethanol production, cell growth, colony forming units, energy status, and fermentation capacity.

### Importance of strain selection on the fermentation of lignocellulosic hydrolysates

Fermenting lignocellulosic hydrolysates and side streams into ethanol or other bulk chemicals is a demanding task for fermenting microorganisms. *S. cerevisiae* is considered the most robust microorganism for industrial scale fermentation in inhibitory media [28]. However, the fermentation performance of different *S. cerevisiae* strains critically depends on the culturing conditions [29-32]. In selecting an appropriate strain, we should consider the metabolic and physiological characteristics of the strain under the processing conditions (T and pH), and with regard to the ethanol, sugar and inhibitor concentrations in the fermentation medium. The two C6 fermenting strains of *S. cerevisiae* evaluated in the present study, namely, Thermosacc and CCUG, represent different strain selection strategies. Thermosacc is specifically designed to cope with certain challenges during fermentation, while CCUG has naturally evolved under the harsh industrial conditions prevailing in a sulphite mill.

**Figure 1:**
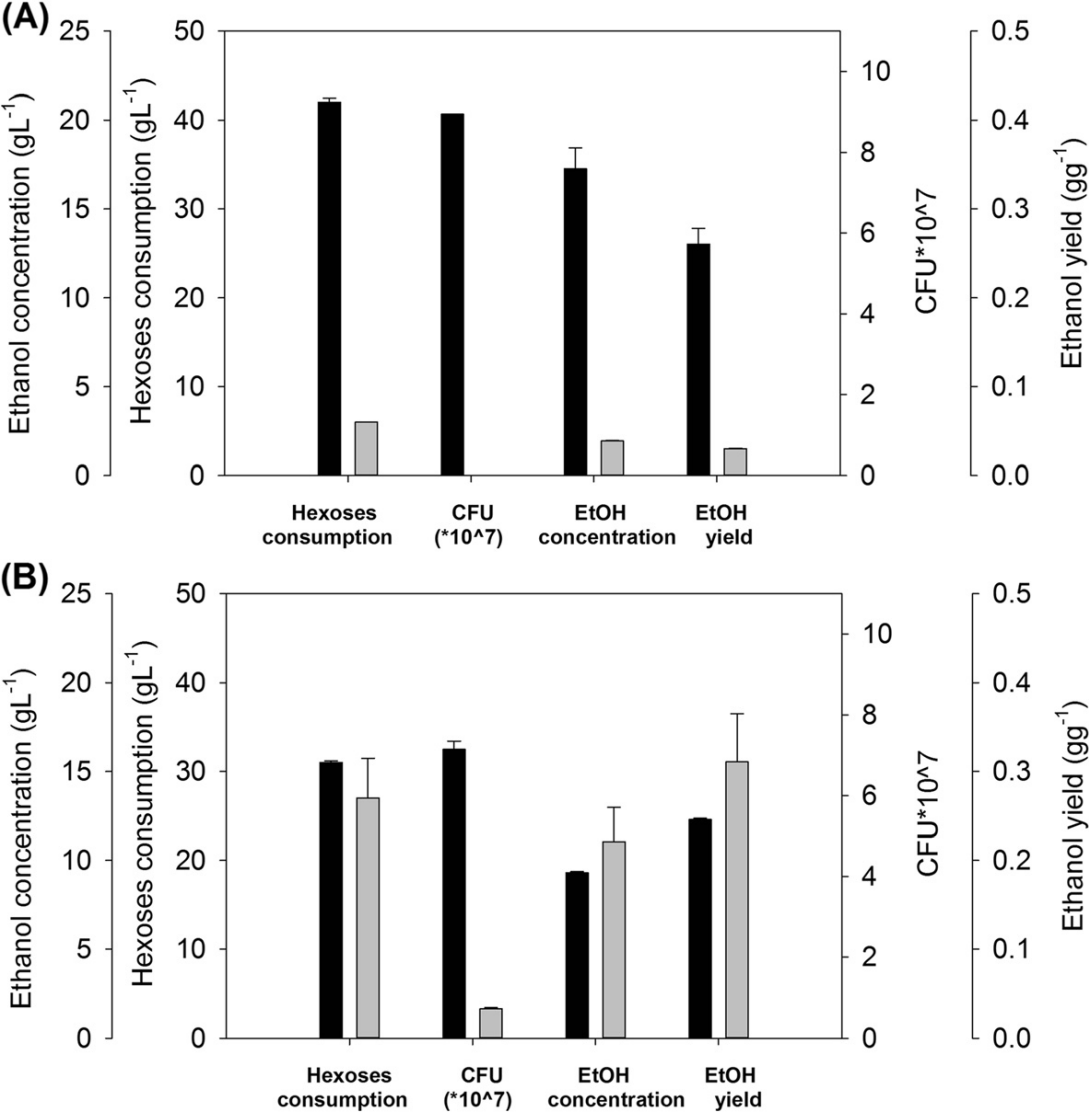
**Hexose consumption, viability and ethanol production after 22 h fermentation.** Fermentation substrate was **(A)** spruce hydrolysate **(B)** spent sulphite liquor. Black and grey bars denote the *S. cerevisiae* strains Thermosacc and CCUG, respectively. Error bars indicate the maxima and minima of two independent fermentations.

As shown in Figure 1A, Thermosacc satisfactorily fermented SH and SSL under the prevailing conditions. After 22 h fermentation, the ethanol concentrations in SH- and SSL-containing media were 17 g L^−1^ and 9 g L^−1^, respectively, corresponding to yields of 0.26 g_ethanol_ and 0.25 g_ethanol_ per g total hexoses. Although Thermosacc growth was limited in terms of cell dry weight (CDW) (data not shown), the viability of the cells (measured by the CFU, Figure 1) was high and remained more or less stable throughout the fermentation period. The strain harvested from the sulphite mill (CCUG) failed to ferment SH, but performed quite well in SSL, achieving an ethanol concentration of 11 g L^−1^ (a yield of 0.31 g_ethanol_ g^−1^ total available hexoses) after 22 h fermentation. These results suggest that the CCUG strain has successfully adapted in the sulphite mill, but loses its adaptive benefits when exposed to a moderately different set of conditions. This hypothesis is consistent with earlier reports that stressed cells continuously and randomly produce genetic variants, which are immortalized as mutations only if they permit cell growth [33,34]. The low level of viable cells after 22 h fermentation in SH indicates that CCUG could not overcome the high toxicity of this foreign medium (Figure 1). However, it should be mentioned that viable cell counts of CCUG were comparably low in SSL, despite the strong fermentation performance in this medium.

### Yeast performance in SH and SSL supplemented with yeast extract

The fermentation performance of yeast is varied by the complexity and variety of substances comprising lignocellulosic materials. The SH used in the present study is a high gravity substrate with 20% WIS content. The production of this hydrolysate also increases the concentration of inhibitory compounds and increases the stress factors affecting yeast performance, including osmolarity [35], sugar concentration [36] and ethanol concentration [37]. The concentrations of inhibitors such as acids, HMF, furfural and phenolics are elevated in SH while sulphite levels are higher in SSL (see Methods).

**Figure 2:**
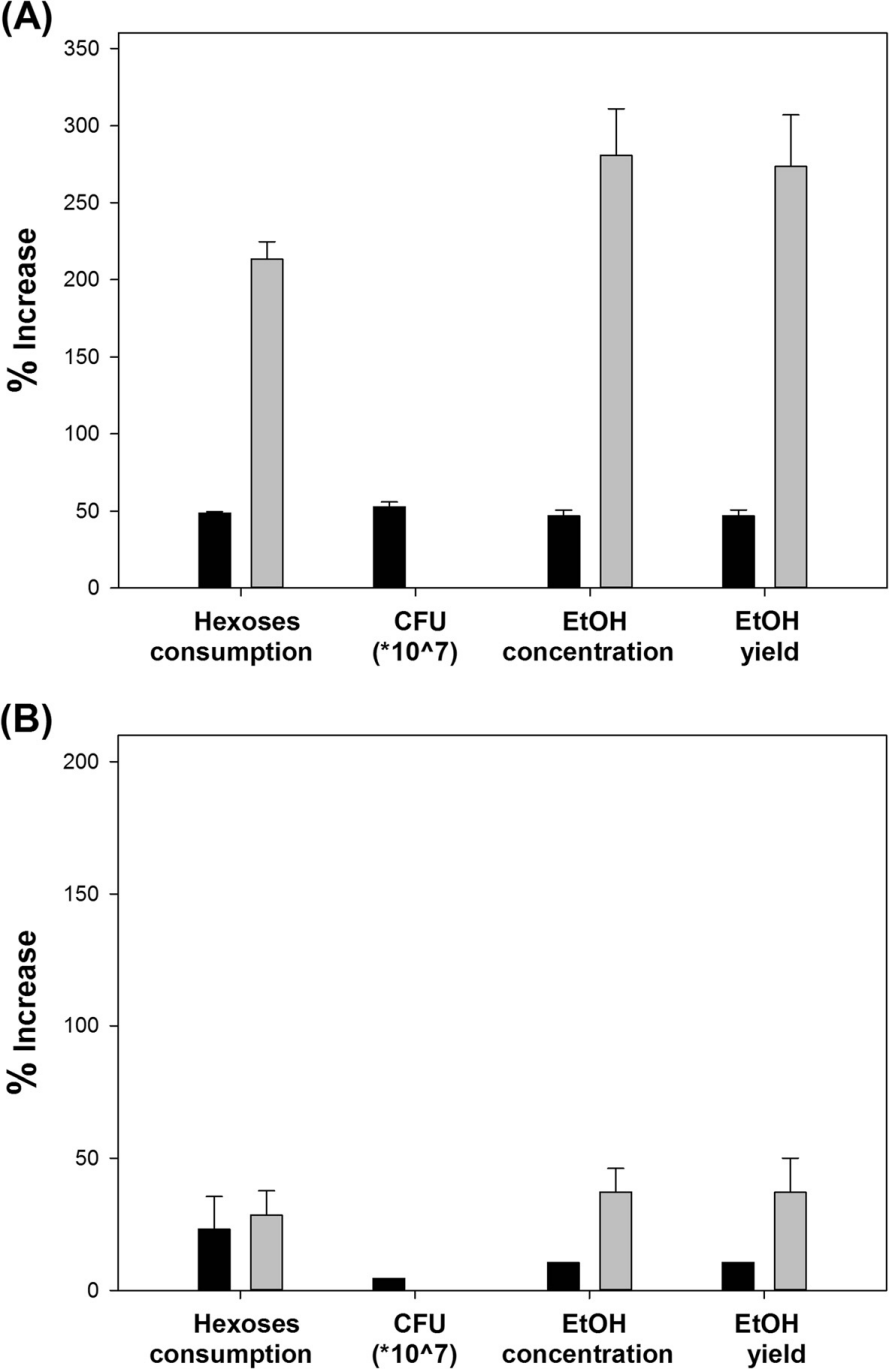
**Increased fermentation performance in media supplemented with yeast extract.** Black and grey bars indicate fermentations with *S. cerevisiae* strains Thermosacc and CCUG 53310, respectively. Fermentation substrate is **(A)** spruce hydrolysate **(B)** spent sulphite liquor. Error bars indicate the maxima and minima of two independent fermentations.

Supplementation with yeast extract (YE) significantly improved the fermentative performance of both yeast strains. Supplemented Thermosacc produced 25.4 g L^−1^ and 10.3 g L^−1^ ethanol from SH and SSL, respectively, corresponding to yields of 0.38 g g^−1^ hexoses and 0.27 g g^−1^ hexoses (based on total fermentable sugar contents in the media). The effect of yeast extract on SH fermentation by CCUG was even more impressive. The sugar uptake was doubled, while the ethanol production almost tripled (Figure 2A). As shown in Figure 2, YE addition affected all of the measured fermentation characteristics to similar extent. Increased ethanol production is partially attributable to the increased cell viability in the presence of yeast extract. However, in the CCUG strain, the higher conversion of hexoses into ethanol indicates that YE additive improved both cell viability and cell metabolism. These findings confirm earlier reports that yeast extract provides cellular building blocks such as amino acids, and also enhances the physiological status of the cells [38,39].

Yeast extract considerably enhanced the biomass formation and viability of Thermosacc in SH-containing medium. The biomass concentration of this organism reached 6 g L^−1^ CDW (data not shown) and the CFU count was 4.73 × 10^7^ (Figure 2A). Nutrient addition also exerted a positive effect in SSL, although to lesser extent than in SH (Figure 2B). The different effects of nutrient addition to the two media probably reflect the higher maintenance requirement of cells fermenting SH. SH contains higher amounts of inhibitory compounds such as phenolics than SSL (5 g L^−1^ in SH versus 1 g L^−1^ in SSL). The biomass production of CCUG was similar in both substrates. In this strain, yeast extract addition exerted a strong stimulatory effect despite the low cell viability. In all cases, the decreased concentrations of residual sugars and increased ethanol formation accompanied a higher biomass concentration, reflecting the improved physiological status of the cells.

The positive impact of supplementing lignocellulosic fermentations with yeast extract is most likely attributable to the complex mixture of vitamins, minerals and free amino nitrogens (FAN) present in yeast extract. All of these compounds are necessary for cell growth, which is correlated with ethanol production [15,16]. Ethanol production may be additionally enhanced by free amino nitrogens, whose potential role in the redox balance may lower the need for glycerol production [13].

### Energy status of yeast cells cultivated in SH and SSL in the absence and presence of yeast extract

Living cells acquire energy through ATP. Besides satisfying a cell’s maintenance energy requirements, ATP is required for growth and biomass formation. Under adverse conditions, maintenance energy requirements escalate to the extent that growth becomes ATP-limited [40]. Concomitant with ATP reduction, AMP levels elevate, with potential decrease in energy charge. Nutrient-poor lignocellulosic substrates contain a cocktail of growth inhibiting substances (reviewed in [3]) that encourage such phenomena. From an ethanol production perspective, low ATP levels are not necessarily disadvantageous, since biomass formation can be restrained while glycolysis and ethanol flux is stimulated [41]. However, ATP levels must remain above a certain threshold to prevent glycolysis inhibition [42].

Cultivated in SH, the Thermosacc strain retained high ATP levels and low AMP levels (Table 1). In contrast, ATP levels temporally declined in the CCUG strain, while AMP accumulated. In SSL-cultivated Thermosacc, ATP levels dramatically decreased after 22 hours fermentation, while AMP levels did not appreciably increase. Again, CCUG was relatively resistant to SSL and retained its ATP levels after 22 hours fermentation (Table 1). These results are consistent with the fermentative performance of the two strains.

**Table 1:** ATP, ADP and AMP concentrations throughout 24 h fermentation

**Strain/condition**	**ATP**			**ADP**			**AMP**			**Energy charge**
Fermentation time (h)	0	4	22	0	4	22	0	4	22	22
Thermosacc/SH	8.06 ± 0.08	8.02 ± 0.17	7.29 ± 0.16	0.18 ± 0.01	0.33 ± 0.06	0.15 ± 0.01	0.16 ± 0.03	0.15 ± 0.00	0.08 ± 0.01	0.98
Thermosacc/SH+	10.94 ± 0.43	11.47 ± 0.04	5.58 ± 0.45	0.55 ± 0.03	0.54 ± 0.01	0.19 ± 0.02	0.14 ± 0.03	0.07 ± 0.03	0.43 ± 0.09	0.92
Thermosacc/SSL	8.83 ± 0.25	5.39 ± 0.38	1.48 ± 0.17	4.76 ± 0.17	2.85 ± 0.55	1.10 ± 0.10	0.23 ± 0.05	0.21 ± 0.02	0.14 ± 0.00	0.75
Thermosacc/SSL+	12.86 ± 0.38	7.01 ± 0.92	1.75 ± 0.63	9.77 ± 0.08	6.40 ± 0.49	2.13 ± 0.57	0.14 ± 0.03	0.39 ± 0.07	0.38 ± 0.05	0.66
CCUG 53310/SH	7.28 ± 0.41	3.46 ± 0.63	2.11 ± 0.05	3.10 ± 0.67	2.4 ± 0.08	1.62 ± 0.19	3.21 ± 0.02	2.83 ± 0.06	2.17 ± 0.20	0.49
CCUG 53310/SH+	13.47 ± 0.24	5.64 ± 1.39	2.15 ± 0.03	6.73 ± 0.06	3.53 ± 0.54	1.67 ± 0.14	0.33 ± 0.01	4.99 ± 0.48	2.68 ± 0.29	0.46
CCUG 53310/SSL	4.95 ± 3.54	N.D.	7.42 ± 6.61	3.40 ± 1.79	N.D.	3.16 ± 2.13	0.05 ± 0.05	N.D.	0.08 ± 0.02	0.84
CCUG 53310/SSL+	5.77 ± 3.11	5.01 ± 0.42	6.26 ± 1.49	5.23 ± 1.65	3.46 ± 0.15	5.07 ± 1.81	0.08 ± 0.01	0.29 ± 0.10	1.59 ± 0.73	0.68

Concentrations are expressed in μmol (g DW)^−1^ unless otherwise stated. SH = Spruce hydrolysate. SSL = Spent sulphite liquor. + indicates that the fermentation has been supplemented with yeast extract.

± indicates the maximum and minimum of two independent fermentations.

Nutrient supplementation exerted little effect on energy charge in either medium. Throughout the SH fermentation, the energy charge in Thermosacc and CCUG was approximately 1 and below 0.5, respectively (Table 1). The low energy charge of CCUG reflects the low viability of the population, which may in turn reflect the high maintenance requirements of this strain. However, CCUG exhibited a higher energy charge in SSL medium, again suggesting that this yeast strain is better adapted to SSL and metabolically equipped for the challenges imposed by this substrate. This hypothesis is supported by the much higher titres and ethanol yields obtained by CCUG in SSL than in SH (Figure 1B). On the other hand, the energy charge of Thermosacc was slightly lower in SSL than in SH (Table 1). This reduction was accompanied by noticeable deterioration in fermentation performance. Hence, under these challenging conditions, maintaining high energetic status is crucial to sustaining a high fermentation rate. However, the absolute ATP levels were not obviously correlated with the rates of glycolysis and ethanol formation.

### Changes in fermentation capacity during cultivation in SH and SSL in the absence and presence of yeast extract

The fermentation capacity of a cell quantifies the ability of the cell to produce ethanol in non-inhibitory nutrient rich media. This measure indicates whether the reduced ethanol production induced by cultivation in low-nutrient lignocellulosic media is restored when the cells are transferred to optimal, non-inhibitory conditions. By investigating how fermentation capacity relates to nutrient supplementation, we can assess whether yeast cells fermenting a lignocellulosic media can be revitalised and consequently reused.

**Figure 3:**
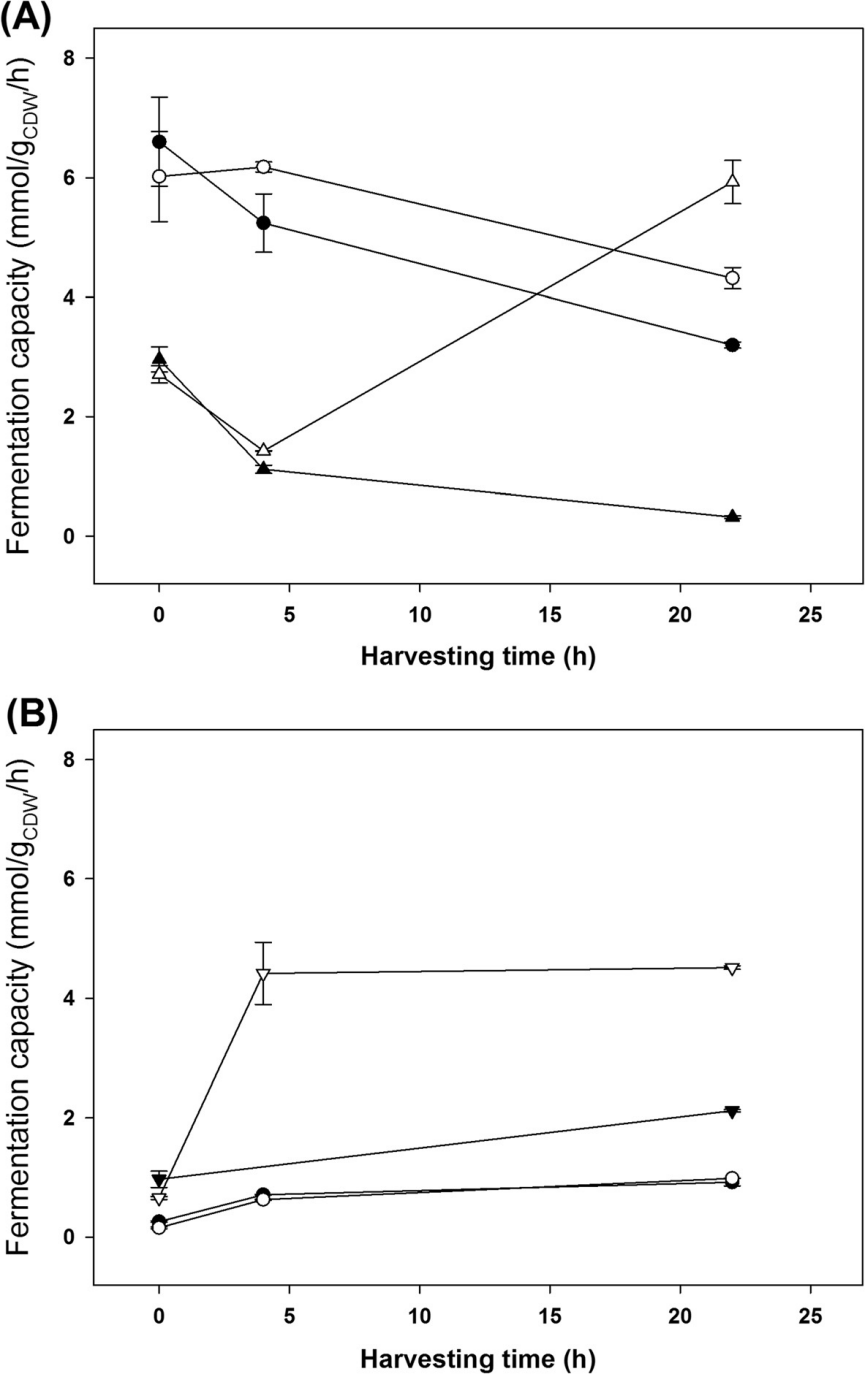
**Fermentation capacity following re-inoculation in YPD media.** Open and closed symbols represent cells harvested from nutrient-supplemented and non-supplemented fermentations, respectively. Circles and triangles denote the *S. cerevisiae* strains Thermosacc and CCUG 53310, respectively. **(A)** Fermentation capacity of cells harvested from spruce hydrolysate. **(B)** Fermentation capacity of cells harvested from spent sulphite liquor. Error bars indicate the maxima and minima of two independent fermentations.

Nutrient supplementation to the SH fermentation enhanced the fermentation capacity of Thermosacc, although this strain demonstrated reasonable fermentation capacity in the absence of additional nutrients (Figure 3A). The CCUG strain harvested from SH fermentations performed poorly when re-inoculated in non-inhibitory nutritional rich media, supporting the hypothesis that CCUG cannot ferment SH. Conversely, Thermosacc demonstrated very poor fermentation capacity in SSL, irrespective of nutrient addition, whereas CCUG responded very positively to nutrient-supplemented SSL. This suggests that CCUG is less affected by the conditions prevailing in SSL than Thermosacc (Figure 3B). The fermentation capacity of CCUG cells harvested from SSL fermentations also increased over time, although levels were initially low. In contrast, the SH fermentation capacity of CCUG generally deteriorated over time. Excepting Thermosacc fermenting SSL, the fermentation capacity was improved in cells harvested from nutrient-supplemented fermentations, indicating that cell viability and revitalizing ability is increased in nutrient-supplemented media.

## Conclusion

This study highlights the importance of selecting a fermentation strain that is well adapted to the prevailing process conditions. The yeast strain CCUG, originally isolated from a sulphite mill, demonstrated stronger performance than Thermosacc in SSL media, while the opposite was observed in SH media. Nutrient supplementation did not moderate this difference, but markedly improved the performance of both strains in both substrates.

## Competing interests

The authors declare that they have no competing interests.

## Authors’ contributions

EJ participated in the design of the study, carried out the fermentations, analysed the results and partially wrote the manuscript. CX participated in the experimental procedure, the HPLC analysis, result analysis and writing of the manuscript. CL conceived the study and participated in analysing the results and writing the manuscript. All authors read and approved the final manuscript.
